# Folic acid conjugated cross-linked acrylic polymer (FA-CLAP) hydrogel for site specific delivery of hydrophobic drugs to cancer cells

**DOI:** 10.1186/1477-3155-12-25

**Published:** 2014-07-15

**Authors:** Jisha Jayadevan Pillai, Arun Kumar Theralikattu Thulasidasan, Ruby John Anto, Devika Nandan Chithralekha, Ashwanikumar Narayanan, Gopalakrishnapillai Sankaramangalam Vinod Kumar

**Affiliations:** 1 Chemical Biology, Rajiv Gandhi Centre for Biotechnology, Thiruvananthapuram-695 014, Poojappura, Kerala, India; 2 Division of Cancer Research, Rajiv Gandhi Centre for Biotechnology, Thiruvananthapuram-695 014, Poojappura, Kerala, India

## Abstract

**Background:**

The hydrogel based system is found to be rarely reported for the delivery of hydrophobic drug due to the incompatibility of hydrophilicity of the polymer network and the hydrophobicity of drug. This problem can be solved by preparing semi-interpenetrating network of cross-linked polymer for tuning the hydrophilicity so as to entrap the hydrophobic drugs. The current study is to develop a folic acid conjugated cross-linked pH sensitive, biocompatible polymeric hydrogel to achieve a site specific drug delivery. For that, we have synthesized a folic acid conjugated PEG cross-linked acrylic polymer (FA-CLAP) hydrogel and investigated its loading and release of curcumin. The formed polymer hydrogel was then conjugated with folic acid for the site specific delivery of curcumin to cancer cells and then further characterized and conducted the cell uptake and cytotoxicity studies on human cervical cancer cell lines (HeLa).

**Results:**

In this study, we synthesized folic acid conjugated cross-linked acrylic hydrogel for the delivery of hydrophobic drugs to the cancer site. Poly (ethyleneglycol) (PEG) diacrylate cross-linked acrylic polymer (PAA) was prepared via inverse emulsion polymerization technique and later conjugated it with folic acid (FA-CLAP). Hydrophobic drug curcumin is entrapped into it and investigated the entrapment efficiency. Characterization of synthesized hydogel was done by using Fourier Transform-Infrared spectroscopy (FT-IR), Transmission Electron Microscopy (TEM), Differential Scanning Calorimetry (DSC). Polymerization and folate conjugation was confirmed by FT-IR spectroscopy. The release kinetics of drug from the entrapped form was studied which showed initial burst release followed by sustained release due to swelling and increased cross-linking. In vitro cytotoxicity and cell uptake studies were conducted in human cervical cancer (HeLa) cell lines.

**Conclusions:**

Results showed that curcumin entrapped folate conjugated cross-linked acrylic polymer (FA-CLAP) hydrogel showed higher cellular uptake than the non folate conjugated form. So this can be suggested as a better delivery system for site specific release of hydrophobic cancer drugs.

## Background

Hydrogels are polymeric networks having three-dimensional configuration capable of imbibing high amounts of water or biological fluids. Their water absorbing property is mainly attributed to the presence of hydrophilic groups such as –OH, −CONH–, −CONH_**2**_–, and –SO_**3**_H in the polymers. Due to the contribution of these groups and domains in the network, the polymer is thus hydrated to different degrees, depending on the aqueous environment and polymer composition [1]. These ionizable functional groups present in it affect its permeability mechanical stability and biocompatibility to a greater extends [2]. Along with that these structures have some common physical properties resembling that of the living tissues, which is attributed to their high water content, soft and rubbery consistency, and low interfacial tension with water or biological fluids [3-5]. The high water content make it soft and wet just like a biological material mimicking the extracellular matrix similar to macromolecular based compound in the human body [6,7]. Hydrogel based drug delivery is a type of controlled delivery system were the gel swell resulting in release of drug from the polymer in a controlled manner. As water penetrate through the polymer chain the glass temperature of the polymer decreases and make the hydrogel rubbery [8]. These hydrogels have highly porous structure which helps incorporation of drug into it. The high water content and high porosity help them easy release of the drug within certain hours to days. The porosity of hydrogel can be tuned to the required size of the drug by the addition of cross linker to it. And thus it help in the controlled release of drug [9,10]. The polymer used for the preparation of hydrogel is of natural, synthetic and semi-synthetic origin. Even though natural polymers have good bioactive properties [11] they are found to have low mechanical strength so we make use of synthetic polymers because of their good mechanical strength and well-defined structure which can be modified to improve the biocompatibility and biodegradability [12,13].

Polyacrylic acid based polymers are one of such ideal candidates for the synthesis of hydrogel system for the controlled drug delivery because of the swelling behavior in aqueous environment [14]. It is a type of pH sensitive polymer [15,16] which shows swelling at higher pH due to the presence of ionizable carboxyl groups in it and can release the drug at neutral pH. The main drawback associated with PAA based drug delivery system is rapid release of drug from it which can be controlled by cross-linking. The cross-linking helps in slow drug release due to small mesh size which can be advantageous for controlled drug delivery applications. Cross-linking also improves the physical properties of the hydrogel including mechanical strength, degradability and diffusivity of drugs from the system. The solubility of polymer in the aqueous environment can also be prevented using cross-linking [17,18].

Curcumin, a naturally occurring yellow coloured polyphenol obtained from the rhizome of the perennial herb *Curcuma longa,* is found to have potent anticancer properties [19]. It inhibit proliferation and induces apoptosis in various cancer cell lines isolated from malignancies like leukemia, breast, lung, prostate and colon tumors [20-23]. Studies were also done in various tumerogenetic models [24-29] and some clinical trials were also done in patients which confirmed the potential of curcumin as a tool for cancer therapy. But its clinical application becomes limited due to poor water solubility, minimal systemic bioavailability, degradation in alkaline pH and photo degradation. So the therapeutic efficacy of curcumin can be increased by incorporating curcumin in a biocompatible polymer which enhances the solubility in aqueous solution and extends the release.

One of the main problems associated with cancer therapy is its unwanted side effect towards normal cells along with the cancer cells. An active targeting strategy can improve the therapeutic efficacy of drugs and reduces the side effects [30-34]. For that we have to modify the polymer nanoparticles with certain ligands that have its specific receptor on cancer cell surface. Folate receptor has been extensively investigated for targeting various tumor cells since it is normally expressed in various types of cancer cells [35-38]. So the cross-linked polymeric hydrogel nanoparticles which were structurally modified with folic acid can help in easy targeting and up taking of drugs by the cancer cells.

The hydrogel based system is found to be rarely reported for the delivery of hydrophobic drug due to the incompatibility of hydrophilicity of the polymer network and the hydrophobicity of drug. This problem can be solved by preparing semi-interpenetrating network of cross-linked polymer for tuning the hydrophilicity so as to entrap the hydrophobic drugs. The current study is to develop a folic acid conjugated cross-linked pH sensitive biocompatible polymeric hydrogel to achieve a site specific drug delivery. For that, we have synthesized a folic acid conjugated PEG cross-linked acrylic polymer (FA-CLAP) hydrogel and investigated its loading and release of curcumin. Here we used inverse emulsion polymerization technique proposed by Vanderhoff et al. for the polymerization of acrylic acid where an aqueous solution of hydrophilic monomer acrylic acid is dispersed in a continuous lipophilic phase with the aid of surfactants to promote the formation of water in oil (W/O) emulsion [39]. The formed polymer hydrogel was then conjugated with folic acid for the site specific delivery of curcumin to cancer cells and then further characterized and conducted the cell uptake and cytotoxicity studies on human cervical cancer cell lines (HeLa).

## Results and discussion

### Synthesis and characterization of FA-CLAP hydrogel

**Figure 1:**
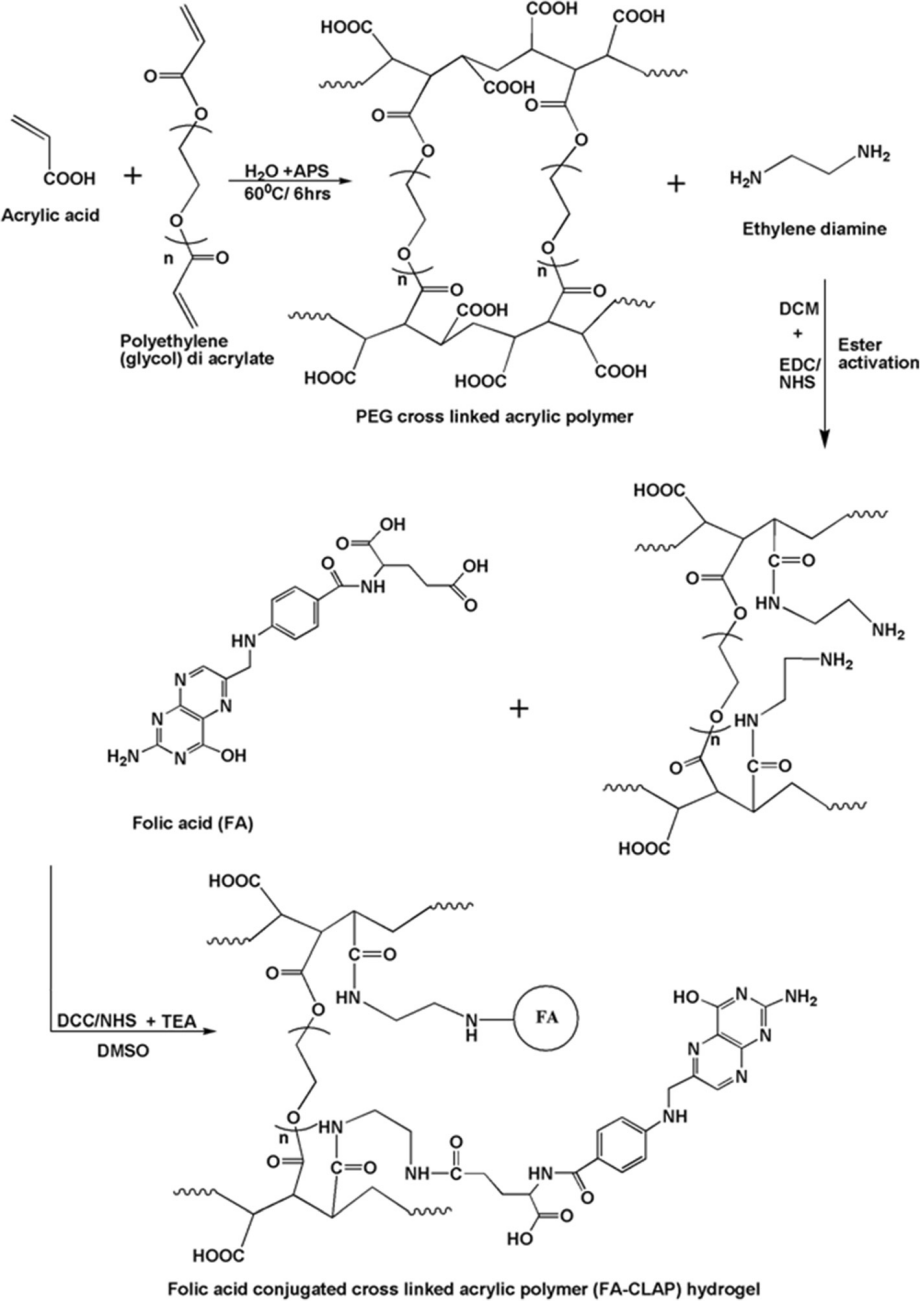
**Scheme of polymer synthesis.** Schematic representation showing the synthesis of folate conjugated cross-linked acrylic polymer hydrogel.

Curcumin loaded folic acid conjugated cross-linked acrylic polymer (FA-CLAP) hydrogel were synthesized successfully using inverse emulsion polymerization technique (Figure 1). Inverse emulsion polymerization is a controllable technique used for the preparation of well- defined nanoparticles. In the present study, we prepared acrylic polymer cross-linked with polyethylene glycol diacrylate by inverse micro emulsion polymerization method which is cross-linked with folic acid through ethylenediamine for targeted delivery of curcumin to cancer cells. Inverse polymerization helps in the easy facilitating of free radical polymerization of acrylic monomer with PEG diacrylate in presence of ammonium persulfate. PEG, a highly biocompatible and hydrophilic polymer with low Tg and polyacrylic acid in the system imparts pH sensitivity. The hydrogel that we prepared displays pH sensitive nature which can be exploited for site specific controlled drug delivery. Folic acid is also conjugated with the hydrogel that helps in the targeted delivery of the drug encapsulated hydrogel towards the cancer cells since many cancer cells are expressing folic acid on its surface and pH sensitivity helps in the swelling of the hydrogel in the required site with the sustained release of drug on that particular site. The percentage swelling in a pH dependant manner was analyzed and given in Additional file 1.

### Characterization

**Figure 2:**
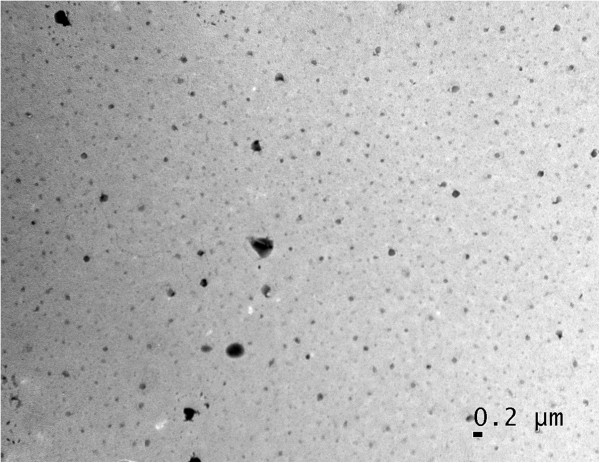
**Transmission electron microscope image of (FA-CLAP).** TEM image of prepared curcumin loaded FA-CLAP hydrogel nanoparticles.

Morphology and the size of the particles were studied using Transmission electron microscopy. The nanoparticles obtained were of size range of 160–190 nm with narrow size distribution (Figure 2). The folic acid conjugated cross-linked polymer hydrogel particle shows a round morphology with nanometric size range. Particle size was found to increase with folic acid conjugation in it.

**Figure 3:**
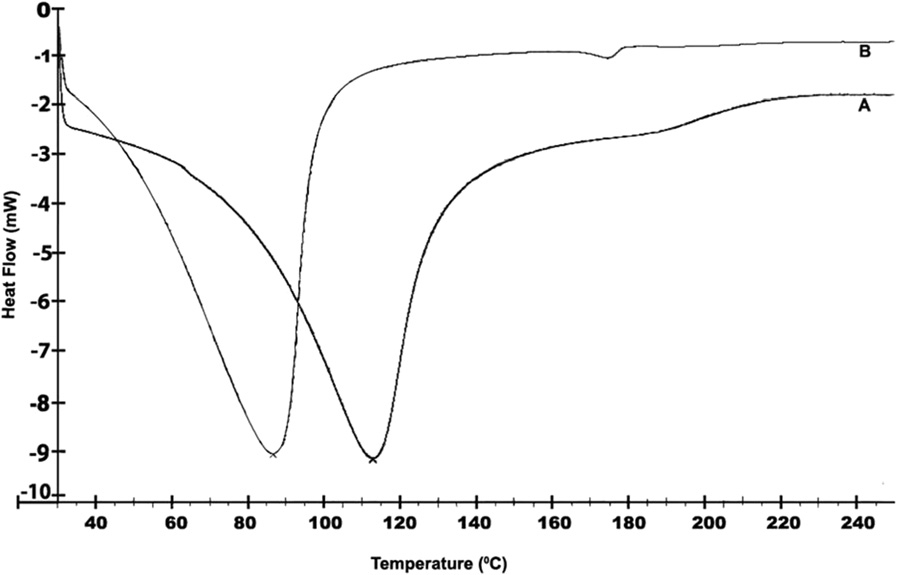
**Differential scanning calorimetry (DSC) of hydrogel.** DSC of samples **(A)** FA-CLAP hydrogel **(B)** Curcumin-entrapped FA-CLAP hydrogel.

Role of size in drug delivery is well known. Optimal size should be needed for a drug delivery particle so that it will not easily leak out of the capillaries and also not easily up taken by the macrophages. Here also we developed a particle which is having a size of about 190 nm that is optimal for the cellular uptake and the folic acid conjugation will help in the active targeted delivery of nanoparticle into the cancer site. Active targeting help in faster accumulation of nanoparticles in the cancer site and help in the release of drug to that particular site [12].DSC was done to find the thermal behavior of the nanoparticles. DSC of folic acid conjugated cross-liked acrylic polymer and curcumin loaded folic acid conjugated cross-linked polymer were done as showed in Figure 3. Folic acid conjugated polymer shows an endothermic peak at 115°C which after loading with curcumin showed a decrease in value (endothermic peak at 85°C) and the nanogel was found to be stable up to 200°C.

The DSC of FA-CLAP hydrogel shows endothermic transition peak at 115°C which can be due to the loss of loose and bound water in the hydrogel [40]. The gel appeared to be thermally stable up to 200°C. Curcumin was loaded to the cross-linked acrylic polymer through physical adsorption by post loading method. And the DSC of curcumin loaded FA-CLAP hydrogel shows an endothermic peak at 85°C. Cross-linking using PEG diacrylate provides hydrophobicity to the acrylic hydrogel which enhances the uptake of curcumin, a hydrophobic drug. Swelling of the polymer occurs at a pH above the pKa of the carboxyl group of acrylic acid. Swelling increases with COO- groups and decreases with increasing cross-links.

**Figure 4:**
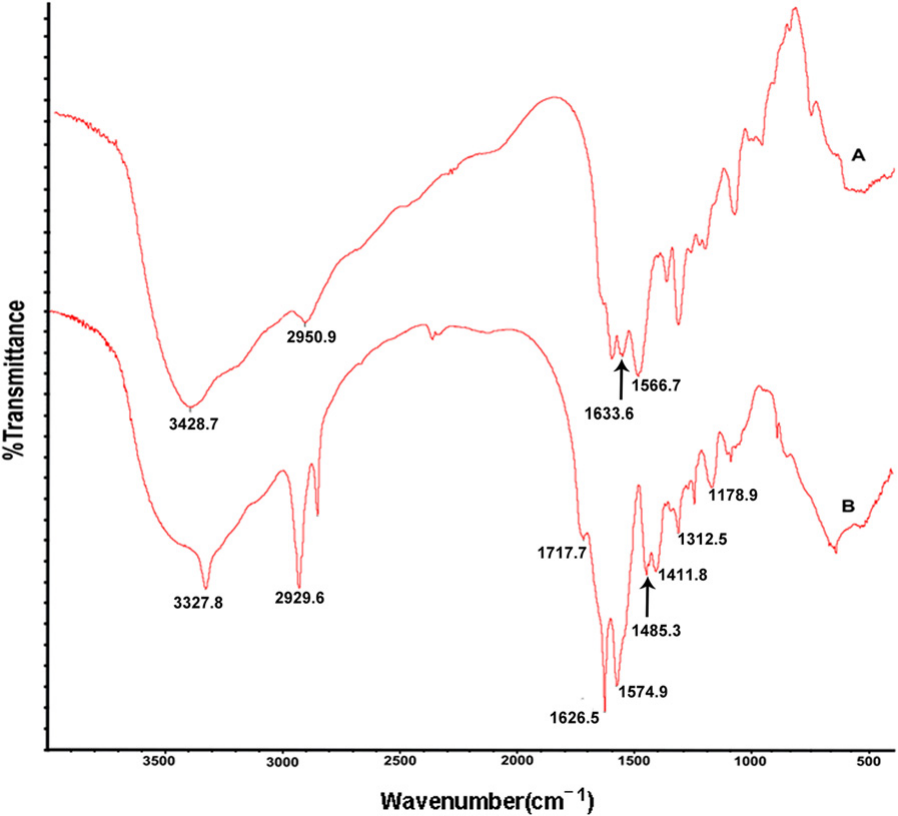
**Fourier transform infra red spectra.** FTIR of the synthesized co polymer: **(A)** Polyacrylic acid with ethylenediamine **(B)** FA-CLAP hydrogel.

The cross-linking and folate conjugation was confirmed by FTIR spectroscopy. FTIR spectroscopy clearly gives idea about cross-linking and conjugation of folic acid into the polymer (Figure 4). FTIR of cross-linked PAA with EDA (ethylenediamine) was shown in Figure 4(A) in which the peak at 3428.7 cm^−1^ may be due to N-H stretching of free amino group in ethylenediamine and peak at 1633.6 cm^−1^and 1566.7 cm^−1^ may be of stretching of amide bond І and II that is formed between ethylenediamine and cross-linked PAA which indicates EDA conjugation with the polymer. Peak at 2950.9 cm^−1^ may be due to aliphatic C-H stretching of poly acrylate. FTIR of FA-CLAP hydrogel was shown in Figure 4(B) in which the absorption band at 2929.6 cm^−1^ is due to asymmetric C-H stretching vibration of folic acid. In addition to this other peaks like 1485.3 cm^−1^ (−C = C- aromatic stretching of phenyl ring) and 1411.8 cm^−1^ (OH deformation of phenyl skeleton) confirm the presence of folic acid in FA-CLAP [41]. Peaks at 1626.5 cm^−1^ and 1574.9 cm^−1^ are due to amide I and amide II band present in FA-CLAP. Peak at 1312.5 cm^−1^ may be due to C = N medium stretching in the pteridine ring of folic acid which all confirmed the folic acid conjugation with ethylenediamine conjugated cross-linked PAA. It shows peak at 1717.7 cm^−1^ which may be due to the presence of free COOH groups in the prepared polymer.

### *In-vitro* drug release

**Figure 5:**
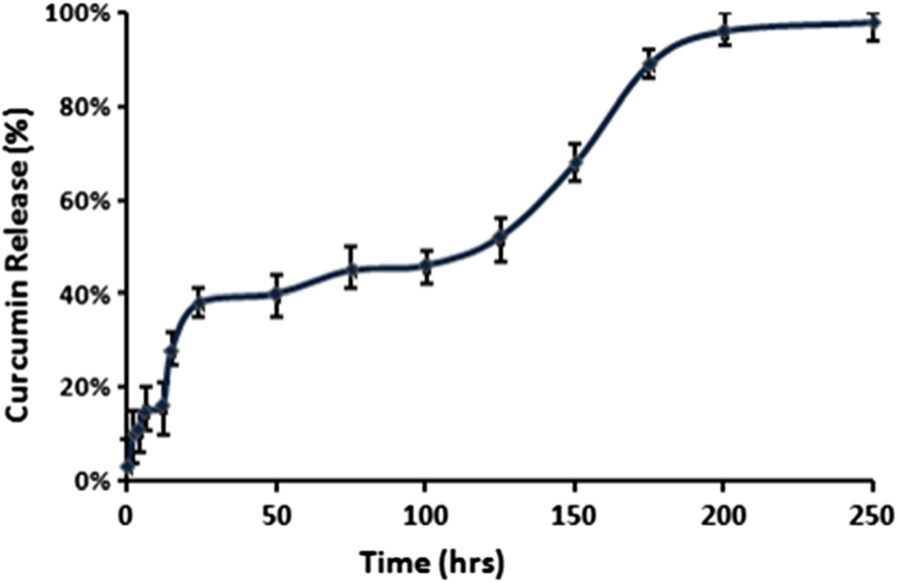
***In vitro *release of curcumin.** Release kinetics of curcumin from FA-CLAP hydrogel.

Curcumin is found to be poorly soluble in water and it form flakes in it. So it is incorporated in a cross-linked polymer hydrogel for delivery to the cancer site. For making targeting more specific, folate conjugation was also done to it. This makes the release of the drug from hydrogel in a controlled manner by the swelling of the gel. Entrapment efficiency of folic acid conjugated cross-linked polymer was found to be 61.2 ± 1.2%. *In vitro* release profile of entrapped drug curcumin from FA-CLAP was done and is shown in (Figure 5). Here the polymer showed an initial release of 10% within 2 hrs and 20-40% release with in 24 hrs (Higuchi diffusion model [42]) because of swelling of hydrogel and thereafter the release occur in slow sustained manner which might be due to the decrease in the swelling because of increased cross-linking. The release was markedly noticeable from 120–200 hours were 50% of the increase in drug release was observed. After 200 hours almost 97% of the drug was released and a steady state was observed. Due to the presence of folic acid, targeted release of drug is possible. Controlled and targeted release of drug from the cross-linked polymer is a desirable property for most of drug delivery applications as it reduces the side effect and increases the bioavailability.

### Cell uptake studies

#### Curcumin entrapped FA-CLAP show better cellular uptake compared to free curcumin

**Figure 6:**
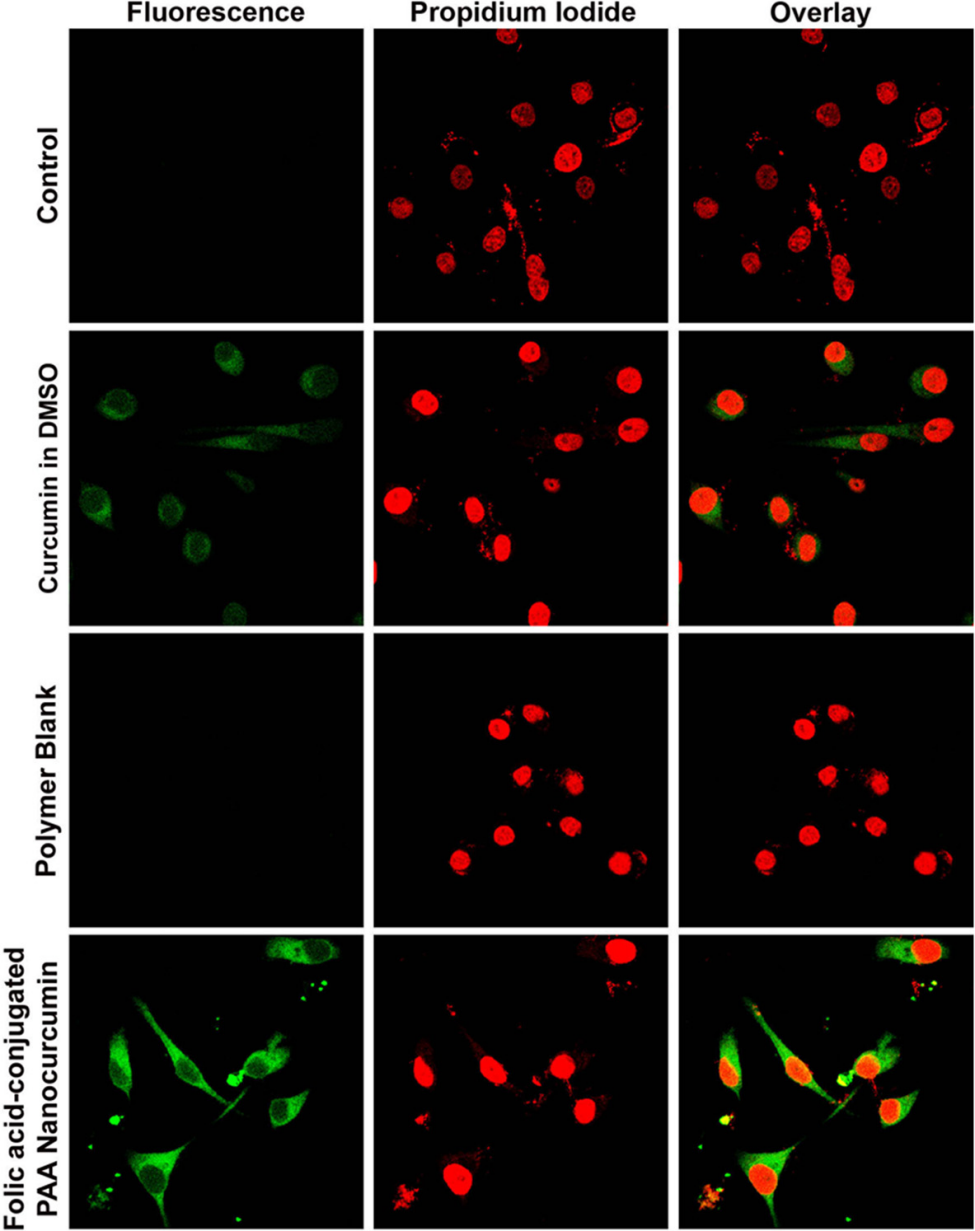
**Cellular uptake image.** Cell uptake of folic acid-conjugated PAA (FA-CLAP) nanocurcumin and free curcumin: HeLa cells were treated with curcumin formulations as mentioned in materials and methods and confocal images were taken in the FITC channel.

We analyzed whether folic acid conjugation to PAA nanocurcumin surface can improve the drug delivery to cancer cells, since cancer cells over-express folate receptors. The internalization of curcumin to the cells was visualized by confocal microscopy and the results indicate that curcumin entrapped FA-CLAP hydrogel show better cell uptake than free curcumin dissolved in DMSO (Figure 6).

### MTT assay

#### Folic acid-conjugated PAA (FA-CLAP) nanocurcumin induce cytotoxicity in HeLa cells

**Figure 7:**
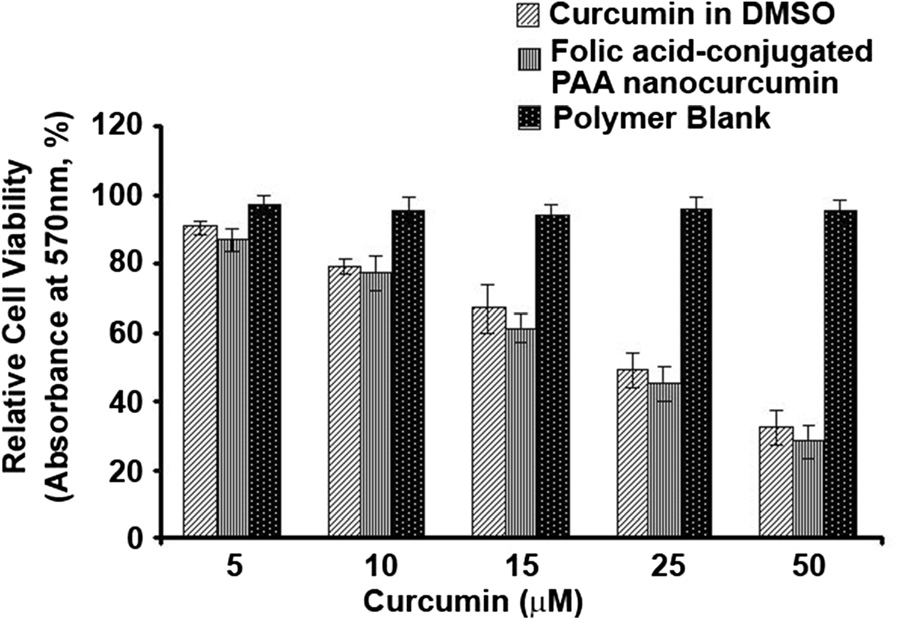
**Cytotoxic effect of folic acid-conjugated PAA nanocurcumin.** HeLa cells were treated with folic acid-conjugated PAA (FA-CLAP) nanocurcumin or curcumin in DMSO. Optical densities were measured and relative cell viability was plotted.

We compared the efficacy of folic acid-conjugated PAA (FA-CLAP) nanocurcumin with its free counterpart in inducing cell death of HeLa cells. HeLa cells were exposed to free curcumin or folic acid-conjugated PAA nanocurcumin (5–50 μM) for 72 h. The nanoformulation imparts high cytotoxicity than free curcumin in dose dependent manner especially for 15 μM, 25 μM and 50 μM. The results indicate that the folic acid-conjugated PAA nanocurcumin (FA-CLAP) show comparatively better efficacy in inducing cytotoxicity of HeLa cells than free curcumin dissolved in DMSO (Figure 7).

### Acridine orange (AO)/ethidium bromide (EB) staining

#### Conjugation of Folic acid on PAA nanocurcumin slightly enhance the apoptotic effect induced by the latter in HeLa cells

**Figure 8:**
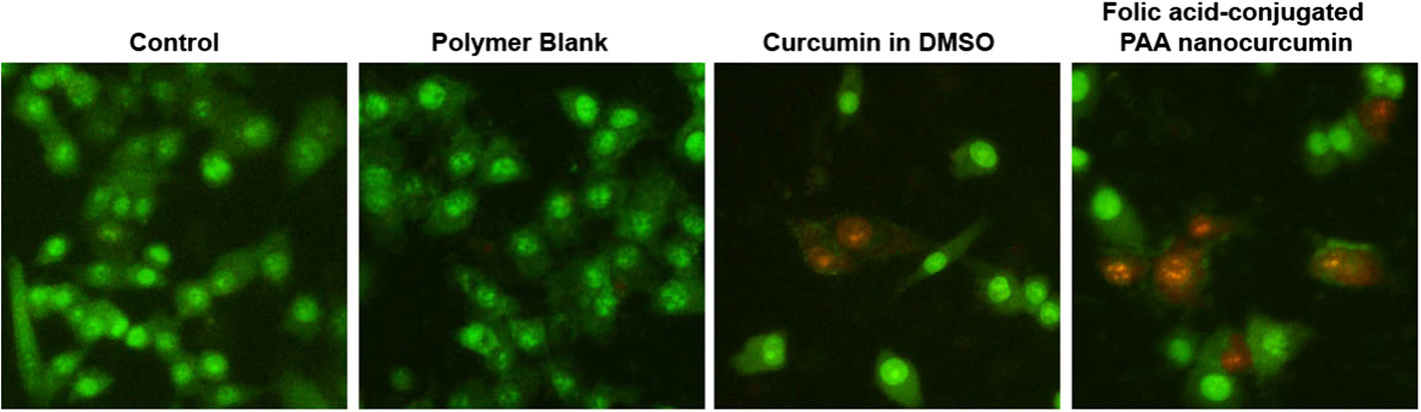
**Fluorescence images of HeLa cells treated by free curcumin or folic acid-conjugated PAA (FA-CLAP) nanocurcumin showing apoptosis.** HeLa cells were treated with different curcumin formulations for 24 h and were stained with acridine orange and ethidium bromide solutions as mentioned in materials and methods and fluorescence images were taken using an inverted fluorescent microscope.

Ability of curcumin in inducing apoptosis of HeLa cells were assessed by AO/EB staining. HeLa cells were exposed to free curcumin or folic acid-conjugated PAA (FA-CLAP) nanocurcumin (25 μM) for 24 h. When treated with AO/EB mixture the untreated cells showed bright green chromatin due to AO staining. The damaged membrane of the apoptotic cells allowed EB to get in, giving red color to the nucleus. In Figure 8 we can vividly observe more number of cells in the red colour (6 out of 16 cells, ~40%) than blank curcumin (2 out of 10 cells, ~20%).The results indicate that conjugation of folic acid on PAA nanocurcumin enhances its apoptotic activity in HeLa cells compared to the free curcumin dissolved in DMSO as assessed by increase in number of EB stained cells (Figure 8).

## Conclusions

Curcumin, which has many medicinal properties mainly, lacks its activity due to its low water solubility. So through this work the delivery of hydrophobic drug curcumin is done by incorporating the drug into cross-linked hydrogel matrix and the cell uptake which was another problem was further enhanced by introducing folic acid into the system. It was found that folic acid conjugated cross-linked hydrogel polymer (FA-CLAP) loaded with curcumin showed better cellular uptake compared to the non-folate hydrogel particles. So this can be used as a better system for the site specific delivery of hydrophobic drugs.

## Methods

### Materials

Acrylic acid (Mw ~ 72), cross-linker poly (ethylene glycol) diacrylate (Mw ~ 238,), Curcumin, Ammonium persulphate (APS), Span 80 (Sorbitan monooleate), Tween 80 (Poly (ethyleneglycol) sorbitan monooleate), 3-(4,5-dimethylthiazol-2-yl)-2, 5-diphenyltetrazolium bromide (MTT), ethylenediamine and folic acid were purchased from Sigma-Aldrich, Germany. Acridine orange and ethidium bromide were purchased from Sigma Aldrich. DMEM was purchased from Life Technologies (Grand Island, NY, USA). MTT was purchased from Calbiochem, Germany and Propidium Iodide from Calbiochem, USA. All other reagents and chemicals were of analytical grade or above, and used without further purification.

### Preparation of folic acid conjugated cross-linked polymeric nanoparticles

#### Preparation of cross-linked acrylic polymer

The cross-linked acrylic polymer (1%) was prepared by inverse emulsion polymerization technique. Emulsification was done by dispersing aqueous phase consisting of 10% acrylic acid, 5% sodium hydroxide and 15% water with continuous lipophilic phase consisting of liquid paraffin (68%), emulsifiers (2%), Span 80 and Tween 80 (75:25 ratio). For cross-linking, 1% of PEG diacrylate was added to the mixture followed by the addition of initiator, ammonium persulfate (APS). The temperature for polymerization was at 60°C for 6 hours. The cross-linked polymeric particles were isolated by centrifugation (10,000 rpm) for 30 min. The isolated polymeric particles were washed several times with hexane and stored for further structural modifications.

#### Conjugation of folic acid to the prepared cross-linked polymeric particles (FA-CLAP)

For the conjugation of folic acid to the prepared cross-linked polymeric particles, first the cross-linked polymeric particle (850 mg. 1.2 eq) has to be treated with ethylenediamine (3.2 ml, 0.65 eq) through carbodiimide chemistry for the availability of free amine group for the binding of activated folic acid to it. Simultaneously folic acid (220 mg, 0.5 eq) has to be ester activated then it is allowed to react with ethylenediamine conjugated cross-linked acrylic hydrogel. A measured amount of this ethylenediamine conjugated cross-linked polymeric particles (550 mg) was dissolved in DMSO. The reaction mixture is kept overnight stirring, for the completion of folic acid conjugation; cross-linked polymeric particles were isolated by centrifugation (10,000 rpm) for 30 min. The isolated folic acid conjugated cross-linked polymeric hydrogel (FA-CLAP) was washed several times and then freeze dried to remove solvent and water. The freeze dried product was stored in vacuum. The polymerization of acrylic acid with PEG diacrylate cross-linking and folic acid conjugation was characterized using DSC and FT-IR spectroscopy. The amount of folic acid conjugated was also estimated (see Additional file 1).

### Drug loading

Loading of curcumin in folic acid conjugated cross-linked polymeric nanoparticles (FA-CLAP) was done by post-polymerization method. 100 mg of the lyophilized powder was dispersed in 10 mL distilled water. Curcumin was dissolved in chloroform and the drug solution in chloroform was added to the polymeric solution with constant vortexing and sonication. The curcumin loaded FA-CLAP hydrogel was then lyophilized to obtain dried powder.

### Characterization of prepared hydrogel nanoparticles

Morphological analysis of the free and curcumin loaded FA-CLAP nanoparticles were then characterized using transmission electron microscopy (TEM, JEOL 1011, and Japan). The samples of the nanoparticle suspension in water milli-Q at 25°C were dropped on to formvar-coated grids and measurements were taken only after the samples were completely dried.

Folic acid conjugation in the parent polymer was confirmed using FTIR spectroscopy. FTIR spectroscopy was performed on a Spectrum 65 (Perkin Elmer). Spectra were recorded between 4000 and 600 cm^−1^ wave number range. Dried samples were mixed with KBr and further compressed in to pellets for making measurements.

Differential Scanning Calorimetry (DSC) was done to analyze the thermal behavior of the FA-CLAP and Curcumin loaded FA-CLAP hydrogel. DSC thermograms obtained were then analyzed using an automatic thermal analyzer system (Pyres 6 DSC, Perkin-Elmer, USA). Samples were placed in standard aluminum pans and heated from 20 to 250°C at a rate of 10°C/minute under constant purging of N_2_ at 10 mL/minute. An empty pan, sealed in the same way as that of the sample, was used as a reference.

### Entrapment efficiency

A known amount of the curcumin loaded folic acid conjugated cross-linked polymer (FA-CLAP) hydrogel nanoparticles were dissolved in methanol and vigorously vortexed to get a clear solution and it is kept for 24 hours, and then filtered through 0.1 μM membrane filter. The absorbance of the filtrate was then taken at 420 nm by using UV absorbance (Perkin Elmer, USA). The entrapped amount of curcumin was then determined by actual entrapment ratio (AER), expressed in terms of amount of curcumin per weight of nanoparticles [43]. Entrapment efficiency can be calculated by the equation

$$\mathrm{Entrapment}\;\mathrm{Efficiency}\;\left(\%\right)=\;\mathrm{AER}\;/\;\mathrm{TER}\;\times \;100$$

(AER = Measured drug wt/ Nanoparticle wt & TER = Initial drug wt/ drug wt & polymer wt). Where (AER) is Actual entrapment ratio and (TER) is Theoretical entrapment ratio. Nanogel wt means the weight of the nanogel with curcumin taken for calculating entrapment efficiency and Initial drug weight means drug initially taken for the entrapment.

### *In vitro* release kinetics

For *in vitro* release study, a known amount of curcumin loaded FA-CLAP were dispersed in 10 mL of P.B.S (pH 7.4) and was then left in a shaking incubator at 37 ± 0.5°C. A known quantity of sample was then withdrawn and replaced with fresh medium in a predetermined time intervals for maintaining the total volume constant. The amount of curcumin released from the hydrogel nanoparticle was then measured using UV spectrophotometer (Perkin Elmer, USA) at 420 nm.

### Cell uptake studies

Cellular uptake of curcumin and folic acid-conjugated PAA (FA-CLAP) nanocurcumin were studied using confocal microscopy. Briefly, 2.0 × 10^4^ HeLa cells were grown on cover slips placed in 24 well plates. After overnight incubation, when the cells attained their morphology, they were treated with curcumin dissolved in dimethyl sulfoxide (DMSO) (25 μM), folic acid-conjugated PAA nanocurcumin (25 μM) was suspended in aqueous medium and blank polymer. After 2 h of incubation the cells were washed with 1X PBS, fixed with PFA and the nuclei were stained with propidium iodide and were mounted using DPX. Cells were examined for intracellular fluorescence of curcumin using confocal laser scanning microscope in the FITC channel (488 nm).

### MTT assay

Cytotoxicity studies of free curcumin and folic acid-conjugated PAA nanocurcumin were carried out in HeLa cells using MTT assay [44]. HeLa cells were seeded (3.0 × 10^3^/well) in a 96-well culture plate and grown for 24 h before the assay. The cells were then treated with different concentrations of curcumin dissolved in DMSO and folic acid-conjugated PAA nanocurcumin (5–50 μM) for 72 h and then 20 μl MTT (5 mg/ml) was added in 80 μl culture medium to each well. After incubating for 2 h at 37°C, cells were lysed using lysis buffer, incubated for 1 h, and the optical densities were measured at 570 nm using a microplate reader (Bio-Rad Laboratories, Hercules, CA). The relative cell viability in percentage was calculated as:

$$\begin{array}{ll} \mathrm{Relative}\;\mathrm{Cell}\;\mathrm{Viability}\; & =\left(A570\;\mathrm{of}\;\mathrm{treated}\;\mathrm{samples}\;/\right)\\ & \;\left(A570\mathrm{of}\;\mathrm{untreated}\;\mathrm{samples}\right)\;\times \;100 \end{array}$$

### Acridine orange (AO)/ethidium bromide (EB) staining

Acridine orange/ethidium bromide (AO/EB) double staining was used to detect apoptosis [45]. Briefly, 5 × 10^3^ cells/well were seeded in a 96-well plate and treated with curcumin in DMSO (25 μM) or folic acid-conjugated PAA (FA-CLAP) nanocurcumin (25 μM) for 24 h. After washing with 1X PBS, the cells were stained with acridine orange (100 μg/ml) and ethidium bromide (100 μg/ml) solutions for 2 min. The cells were then washed with 1X PBS, viewed under an inverted fluorescent microscope (Nikon Eclipse, TE-300) and were photographed.

## Competing interests

The authors declare that they have no competing interests.

## Authors’ contributions

JPG synthesized, characterized the polymer and nanoparticles. DNC and NA have written the final manuscript. AKTT had done the biological experiments. RJA participated in evaluation of the biological experiments and supplied information for writing the final manuscript. GSVK planned the whole work and corrected the manuscript. All authors read and approved the final manuscript.

## Supplementary Material

Additional file 1 Supplementary information. Click here for file
