# Supplementary material for: Folic acid conjugated cross-linked acrylic polymer (FA-CLAP) hydrogel for site specific delivery of hydrophobic drugs to cancer cells
Source: J Nanobiotechnology. 2014 Jul 15;12:25. doi: 10.1186/1477-3155-12-25 (PMC4107481; doi:10.1186/1477-3155-12-25)
Supplement: Additional file 1 — Supplementary information. [file 1477-3155-12-25-S1.doc]

# Supplementary information of

# Folic acid conjugated cross-linked acrylic polymer (FA-CLAP) hydrogel for site specific delivery of hydrophobic drugs to cancer cells

**Jisha J Pillai ,^a^ Arun Kumar T Thulasidasan^b^, Ruby John Anto^b^, Devika Nandan C^a^ , N Ashwanikumar^a^ and G.S.Vinod Kumar^a*^**

*^a^Chemical Biology, Rajiv Gandhi Centre for Biotechnology, Poojappura, Thiruvananthapuram-695 014, Kerala, India.*

*^b^Division of Cancer Research, Rajiv Gandhi Centre for Biotechnology, Poojappura,*

*Thiruvananthapuram-695 014, Kerala, India.*

**Swelling studies**

The gravimetric method was used to study the swelling behavior of FA-CLAP as a function of pH. A known quantity of FA-CLAP and unconjugated CLAP was measured and immersed in an excess quantity of PBS with pH 2.2 and in PBS of pH 7.4 for 24 hours to attain the swelling equilibrium. The weight of the swollen cross linked polymers was measured after excess solvent was removed. The percentage swelling (%S) was calculated as follows

Percentage of swelling = (Ws - Wd)/Wd × 100

where Ws is the weight of the swollen polymer and Wd is the weight of the dried polymer. Results were shown below.

| **Table 1**. Swelling behavior of the hydrogel   |  | | | | --- | --- | --- | | Sample | | | | **Swelling behavior at pH 7.4 (g/g of hydrogel) Swelling behavior at pH 2.2 (g/g of hydrogel)** |  |  | | | FA-CLAP | 19.3 ± 0.94 | 16.1 ± 1.15 | |  |  |  | | | | | | |
| --- | --- | --- | --- | --- | --- | --- | --- | --- | --- | --- | --- | --- | --- | --- | --- | --- | --- | --- | --- | --- |
|  |  |  |  |  |  |
|  |  |  |  |  |  |
|  |  |  |  |  |  |

**Determination of folic acid in FA-CLAP hydrogel**

The folic acid conjugated to FA-CLAP hydrogel was determined by the UV-Visible spectral analysis as follows. Weighed amount of FA-CLAP conjugate was dissolved in Phosphate Buffered Saline (PBS) of pH =7 and 0.1mg/ml of protease solution from Bovine pancreas (Sigma, Germany). The proteases hydrolyse the amide bond in FA-CLAP between folic acid and polymer. The resulting solution was incubated to 72 hours at 37^0^C. Then the solution was centrifuged at 2000 rpm and folic acid was quantified by measuring absorbance at dual wavelengths namely 280 nm and 340 nm. The conjugation efficiency was found to be 82.41 ± 2.3 %.


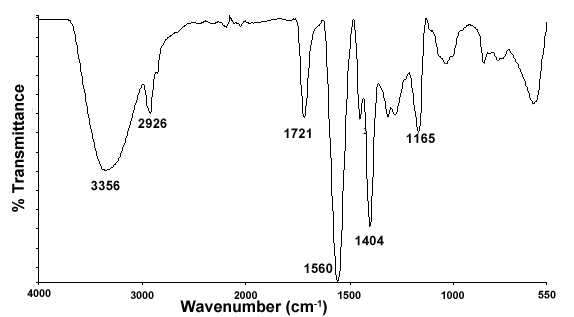


**Fig S1**- FT-IR spectrum of Blank CLAP polymer without folic acid
